# Assessment of the proximity between the mandibular third molar and inferior alveolar canal using preoperative 3D-CT to prevent inferior alveolar nerve damage

**DOI:** 10.1186/s40902-015-0030-4

**Published:** 2015-09-17

**Authors:** Byeongmin Lee, Youngju Park, Janghoon Ahn, Jihyun Chun, Suhyun Park, Minjin Kim, Youngserk Jo, Somi Ahn, Beulha Kim, Sungbae Choi

**Affiliations:** 1 Department of Oral and Maxillofacial Surgery, Kangnam Sacred Heart Hospital, College of Medicine, Hallym University, 1, Singil-ro, Yeongdeungpo-gu, Seoul 150-950 Korea; 2 Department of Orthodontics, Kangnam Sacred Heart Hospital, College of Medicine, Hallym University, 1, Singil-ro, Yeongdeungpo-gu, Seoul 150-950 Korea

**Keywords:** Spiral CT, Third molar, Tooth extraction, Inferior alveolar nerve

## Abstract

**Background:**

The inferior alveolar nerve (IAN) may be injured during extraction of the mandibular third molar, causing severe postoperative complications. Many methods have been described for evaluating the relative position between the mandibular third molar and the inferior alveolar canal (IAC) on panoramic radiography and computed tomography, but conventional radiography provides limited information on the proximity of these two structures. The present study assessed the benefits of three-dimensional computed tomography (3D-CT) prior to surgical extraction of the mandibular third molar, to prevent IAN damage.

**Methods:**

This retrospective study included 4917 extractions in 3555 patients who presented for extraction of the mandibular third molars. The cases were classified into three groups, according to anatomical relationship between the mandibular third molars and the IAC on panoramic radiography and whether 3D-CT was performed. Symptoms of IAN damage were assessed using the touch-recognition test. Data were compared using the chi-square test and Fisher’s exact test.

**Results:**

Among the 32 cases of IAN damage, 6 cases were included in group I (0.35 %, *n* = 1735 cases), 23 cases in group II (1.1 %, *n* = 2063 cases), and 3 cases in group III (0.27 %, *n* = 1119 cases). The chi-square test showed a significant difference in the incidence of IAN damage between groups I and II. No significant difference was observed between groups I and III using Fisher’s exact test. In the 6 cases of IAN damage in group I, the mandibular third molar roots were located lingual relative to the IAC in 3 cases and middle relative to the IAC in 3 cases. The overlap was ≥2 mm in 3 of 6 cases and 0–2 mm in the remaining 3 cases. The mean distance between the mandibular third molar and IAC was 2.2 mm, the maximum distance 12 mm, and the minimum distance 0.5 mm. Greater than 80 % recovery was observed in 15 of 32 (46.8 %) cases of IAN damage.

**Conclusions:**

3D-CT may be a useful tool for assessing the three-dimensional anatomical relationship and proximity between the mandibular third molar and IAC in order to prevent IAN damage during extraction of mandibular third molars.

## Background

The inferior alveolar nerve (IAN) may be injured during extraction of the mandibular third molar, which often results in severe complications for patients [1]. The resulting neurological complications are closely correlated with the anatomical proximity of the IAN, patient age, the root curvature, and the surgical procedure employed to remove distal bone [2–4]. Therefore, many studies recommend preoperative imaging, such as panoramic radiography, standard periapical radiography, computed tomography, and scenography, before surgically removing an impacted mandibular third molar [5–13]. Many methods have been described for evaluating the relative position between the mandibular third molar and the inferior alveolar canal (IAC) on panoramic radiography and tomography [14–19]. However, conventional panoramic radiography provides only limited information on the anatomical morphology of the mandibular third molar and its anatomical relationship to the IAC.

Three-dimensional computed tomography (3D-CT) may be ideal to evaluate the close relationship between the third molars and IAC because it provides a three-dimensional view. Many studies report that the proximity between the third molars and IAC is an important factor in determining the risk of IAN damage after extraction of the mandibular third molar [2, 4]. Therefore, in the present study, we evaluated the usefulness of 3D-CT in preventing IAN damage during surgical removal of the mandibular third molars by determining its proximity to the IAC.

## Methods

A total of 4917 extractions in 3555 patients were included in this retrospective study. All extractions were performed by the oral and maxillofacial surgeons at our hospital. All images were acquired using a panoramic radiographic machine (Auto III N CMR, Asahi Roentgen Ind. Co., Japan) and a 3D-CT unit (Somatom sensation 64 channel, Siemens Ind. Co., Germany). All cases in this study were classified into three groups, according to anatomical relationship between the mandibular third molars and the IAC on the panoramic view and whether a 3D-CT image were acquired (Table 1). Patients in group I exhibited an overlap between the mandibular third molar and IAC on panoramic radiography and underwent spiral 3D-CT. Patients in group II also exhibited an overlap, but they did not undergo spiral 3D-CT. Patients in group III did not exhibit an overlap on panoramic radiography, so they did not undergo spiral 3D-CT. The study protocol was reviewed and approved by the Kangnam Sacred Heart Hospital Institutional Review Board (2013-12-108).

**Table 1:** Classification of cases (Y: yes, N: no)

	Overlapping^a^	3D-CT
Group I	Y	Y
Group II	Y	N
Group III	N	N

^a^Mandibular third molar on IAC on panoramic view

**Fig. 1:**
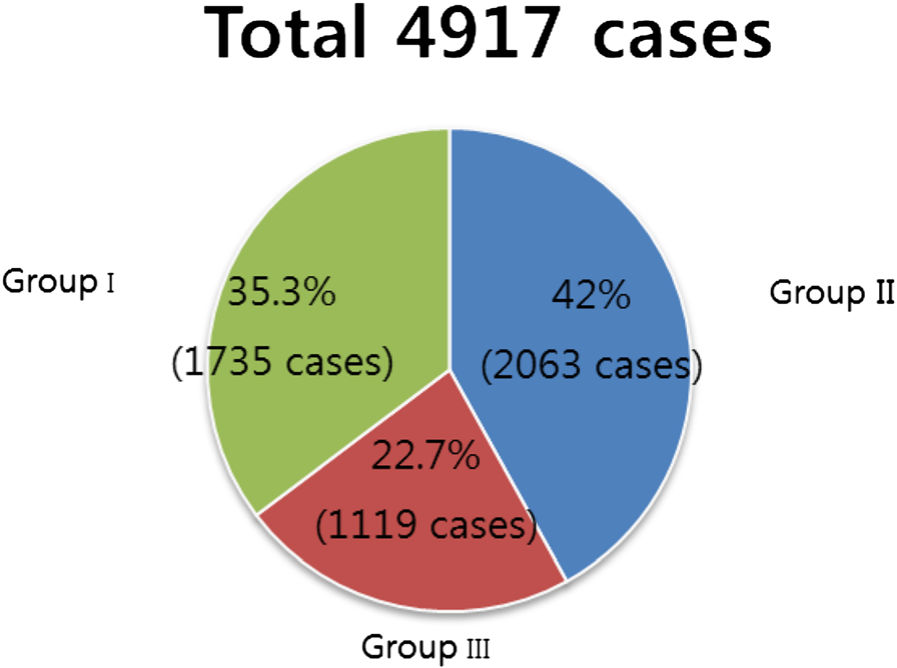
Group-specific distribution of total cases

**Fig. 2:**
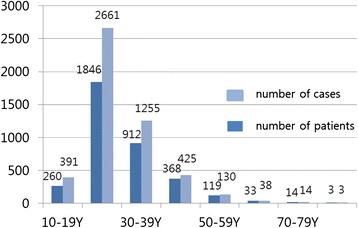
Age-specific distribution of total cases

**Fig. 3:**
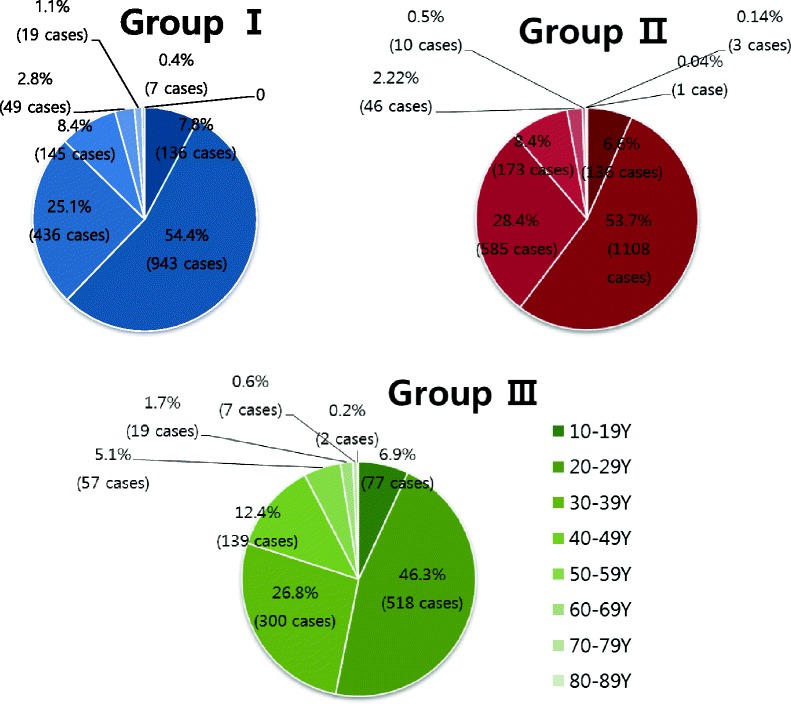
Age-specific distribution per each group

A total of 1735 cases (35.3 %) were classified into group I, 2063 cases (42 %) into group II, and 1119 cases (22.7 %) into group III (Fig. 1). The age distributions are summarized in Figs. 2 and 3. A total 391 of 4917 cases (7.95 %) were aged 10–19 years; 2661 cases (54.19 %), 20–29 years; 1255 cases (25.5 %), 30–39 years; 425 cases (8.6 %), 40–49 years; 130 cases (2.6 %), 50–59 years; 38 cases (0.77 %), 60–69 years; 14 cases (0.28 %), 70–79 years; and 3 cases (0.06 %) were age 80–89 years.

**Fig. 4:**
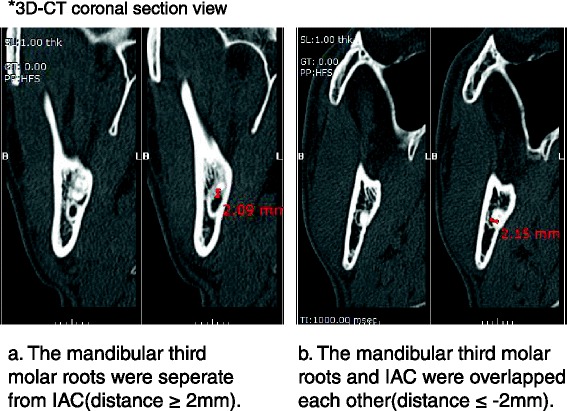
Measuring a distance on mandibular third molar and IAC by Pi-view STAR program

The distance between the mandibular third molar and IAC was measured using the Pi-view STAR program (INFINITT, Seoul, Korea) by a single examiner. The examiner marked a dot on each molar and the adjacent IAC, and the shortest distance between them was measured on the coronal 3D-CT image (Fig. 4).

The touch-recognition test, which measures subjective neurologic symptoms, was performed 1–2 days and 7–10 days postoperatively at the lower lip, chin, gingiva, and tongue to detect IAN or lingual nerve damage. Data were analyzed using the chi-square test and the Fisher’s exact test at a threshold *p* value of 0.05. All statistical analyses were performed using SPSS software version 17.0 (SPSS, Chicago, IL, USA).

## Results

IAN and lingual nerve damage occurred in 32 and 12 of 4917 cases, respectively (total 43 of 4917 cases; Table 2). Hypoesthesia was observed in all cases with IAN damage, but anesthesia did not occur in any case.

**Table 2:** Incidence of lingual nerve damage and IAN damage of each group

	Lingual nerve damage	IAN damage	IAN damage rate (%)
Group I (1735 cases)	3 cases	6 cases	0.35
Group II (2063 cases)	6 cases	23 cases	1.1
Group III (1119 cases)	3 cases	3 cases	0.27
Total (4917 cases)	12 cases	32 cases	0.65

**Fig. 5:**
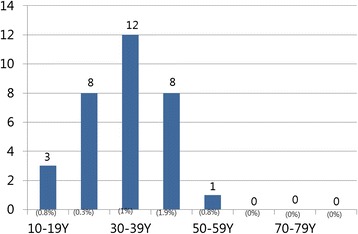
Age-specific distribution of IAN damage cases and IAN damage rate

**Fig. 6:**
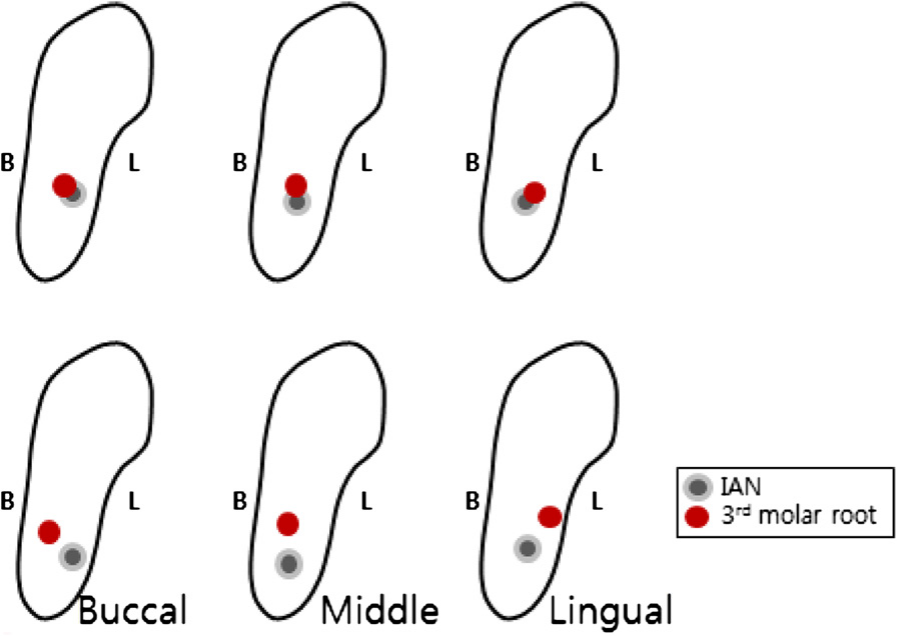
Relative position of mandibular third molar to IAC in group I

**Fig. 7:**
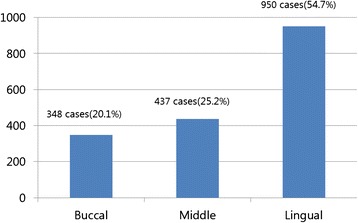
Relative position of mandibular third molar to IAC in group I

The age distribution for the cases of IAN damage was as follows: 3 cases aged 10–19 years; 8 cases 20–29 years; 12 cases 30–39 years; 8 cases 40–49 years; 1 case 50–59 years; and none 60 years and older (Fig. 5). Among the 32 cases of IAN damage, 21 of 32 (65.6 %) cases were right-sided, and 11 of 32 (34.4 %) were left-sided. In group I (*n* = 1735 cases), the anatomical relationship between the overlapping mandibular third molar and IAC was as follows: the mandibular third molar roots were located buccal relative to the IAC in 348 (20.1 %) cases; middle relative to the IAC in 437 (25.2 %) cases; and lingual relative to the IAC in 950 (54.7 %) cases (Figs. 6 and 7).

**Fig. 8:**
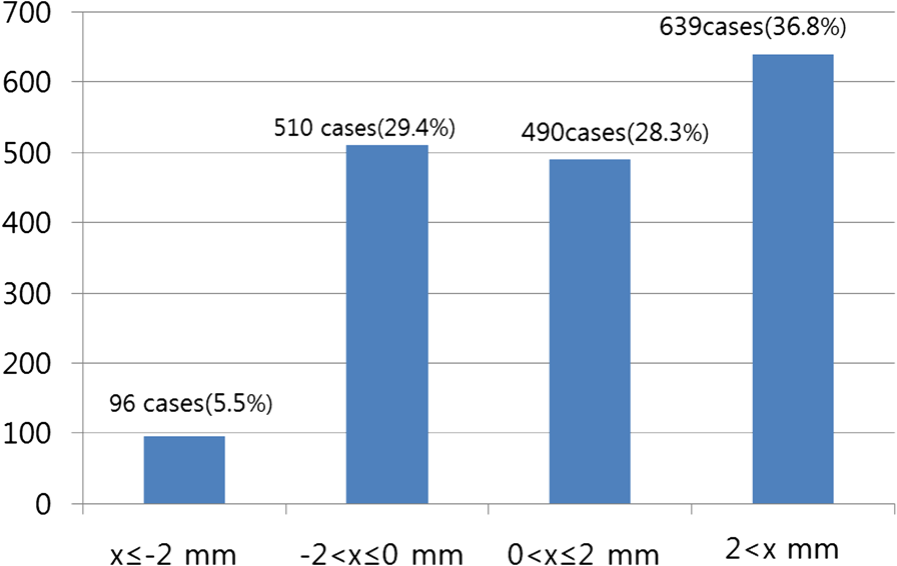
The distance from mandibular third molar to IAC in group I

Figure 8 shows the distances between the mandibular third molar and IAC. In 96 of 1735 (5.5 %) cases, the IAC and third molar root overlapped more than 2 mm, and in 510 cases (29.4 %), they overlapped between 0 and 2 mm. In 490 of 1735 cases (28.3 %), the IAC and mandibular third molar were between 0 and 2 mm apart, and in 639 cases (36.8 %), they were separated by greater than 2 mm. In some images, the mandibular third molar roots appeared to overlap on panoramic radiography, but on 3D-CT, the IAC and third molar roots were separate from each other. In the 6 cases of IAN damage in group I, the mandibular third molar roots were located lingual relative to the IAC in 3 cases, and middle relative to the IAC in 3 cases. The distance between the mandibular third molar and IAC was also investigated in these 6 cases. Three cases showed an overlap of 2 mm or greater, and the remaining 3 cases showed an overlap measuring from 0 mm to less than 2 mm.

In group III, the mean distance between the mandibular third molar and the IAC was 2.2 mm. The maximum distance was 12 mm, and the minimum distance was 0.5 mm.

The recovery from IAN and lingual nerve damage was also assessed. Six of 11 (54.5 %) cases of lingual nerve damage recovered more than 80 %. Greater than 80 % recovery was observed in 15 of 32 (46.8 %) cases of IAN damage (Table 3).

**Table 3:** Recovery of inferior alveolar nerve damage and lingual nerve damage

Recovery of nerve damage	Lingual nerve damage	IAN damage
80 % ≤ *x* ≤ 100 %	6 cases (54.5 %)	15 cases (46.8 %)
60 % ≤ *x* < 80 %	2 cases (18.2 %)	3 cases (9.4 %)
40 % ≤ *x* < 60 %	0 case (0 %)	6 cases (18.8 %)
*X* < 40 %	1 case (9.1 %)	0 case (0 %)
No visit	2 cases (18.2 %)	8 cases (25 %)
Total	11 cases (100 %)	32 cases (100 %)

## Discussion

In our study, both panoramic radiography and 3D-CT were used to evaluate the anatomic relationship between the mandibular third molar and the IAN. The mean incidence of IAN damage during extraction of the mandibular third molar was 0.65 % in our study (Table 2). The incidence of IAN damage varies among previous studies. Carmichael and McGowan [5] reported that the incidence of IAN damage was 0.4–5.5 %; in most cases, IAN damage recovered spontaneously, but permanent paresthesia persisted in less than 1 % of cases. Bataineh [18] concluded that the incidence of IAN damage was 0.4–8.4 % based on review of several previous retrospective studies.

In our study, the mandibular third molar root tended to be located lingually to the IAC (Fig. 8). Kaeppler [12] found that the IAC was located buccal relative to the mandibular third molar roots and recommended performing CT in addition to conventional panoramic radiography for a more accurate anatomic diagnosis. Similarly, Pawelzick et al. [13] recommended performing volumetric CT to assess the location of the mandibular third molar and IAC three-dimensionally.

In the present study, the incidence of IAN damage was 0.35 % in group I, 1.1 % in group II, and 0.27 % in group III. Nakagawa et al. [20] asserted that it was important to know the anatomical proximity between the mandibular third molar and IAC before molar extraction as understanding this proximity was critical in preventing IAN damage.

The IAN damage rate was highest in patients aged 40–49 years. Patients aged 60 years and older were not subjected to detailed analysis in our study because none experienced IAN damage (Fig. 5). Few studies have examined patient age as a risk factor of IAN damage, though Valmaseda et al. [2] reported that the risk of IAN damage increased as patients aged. Two theories have been proposed to explain this phenomenon. Potentially, surgical trauma may be more severe in older patients, and alternatively, healing may be poorer in older patients than in younger patients. However, Kipp et al. [21] stated that there was no relationship between age and IAN damage.

In this study, the incidence of IAN damage was 0.35 % in group I and 1.1 % in group II and, according to the chi-square test, differed significantly between these two groups (*P* < 0.05). This result shows that using 3D-CT can reduce the incidence of IAN damage in the case of overlap between the mandibular third molar and IAC on panoramic radiography.

There was no statistically significant difference in the incidence of IAN damage between group I (0.35 %) and group III (0.27 %) according to Fisher’s exact test (Table 4). This is that extraction can be performed in group I with similar incidence rate of IAN damage to group III if extraction was performed after careful identification about the locations of the compared IAC and root.

**Table 4:** Statistical analysis

	Statistical analysis	*p* value
Comparison of group I and group II	The chi-square test	0.007^*^
Comparison of group I and group III	Fisher’s exact test	1.0^**^

^*^Statistically significant (*P* < 0.05)

^**^Statistically not significant (*P* < 0.05)

Collectively, these results indicate that 3D-CT may be a useful diagnostic tool in preventing IAN damage in patients undergoing extraction of the mandibular third molar.

In addition, the removal of impacted teeth in orthognathic surgery is well-documented and recommended at least 9 to 12 months prior to the planned orthognathic surgery [22]. The findings in this study can be used to reduce IAN damage during mandibular third molar extraction before orthognathic surgery and the fracture line design using 3D-CT has to be additionally studied for orthognathic surgery with unerupted third molars.

## Conclusions

Preoperative radiographic examination is necessary to determine the relationship between the mandibular third molar and IAC as it helps prevent IAN damage during extraction of the mandibular third molar. Based on the current findings, we conclude that 3D-CT imaging may be a useful tool in assessing the anatomical proximity between the mandibular third molar and IAC in three-dimensions and preventing IAN damage in patients undergoing extraction of the mandibular third molar.

## Abbreviations

3D-CT: three-dimensional computed tomography
IAC: inferior alveolar canal
IAN: inferior alveolar nerve
